# Supplementary material for: Increase in Vascular Function Parameters According to Lifestyles in a Spanish Population without Previous Cardiovascular Disease—EVA Follow-Up Study
Source: Nutrients. 2023 Oct 30;15(21):4614. doi: 10.3390/nu15214614 (PMC10648779; doi:10.3390/nu15214614)
Supplement: Supplementary file 1 [file nutrients-15-04614-s001.zip › nutrients-2670963-supplementary.pdf]

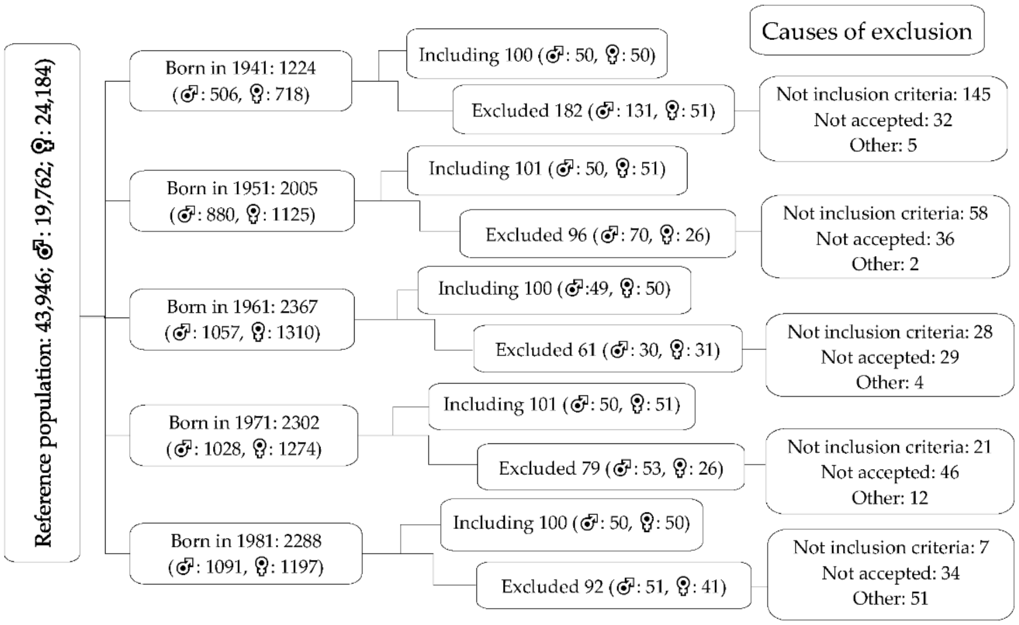

Figure S1. EVA study flowchart. It indicates the reference population by age group of the total population and by sex, the subjects included and excluded and the main causes of exclusion. 259 subjects did not meet inclusion criteria. 177 did not agree to participate in the study and 74 subjects could not be reached because they had changed their address or telephone number. The replenishment rate was 35.4 per cent and the response rate was 64.6 per cent. ♂:Males; ♀:women.

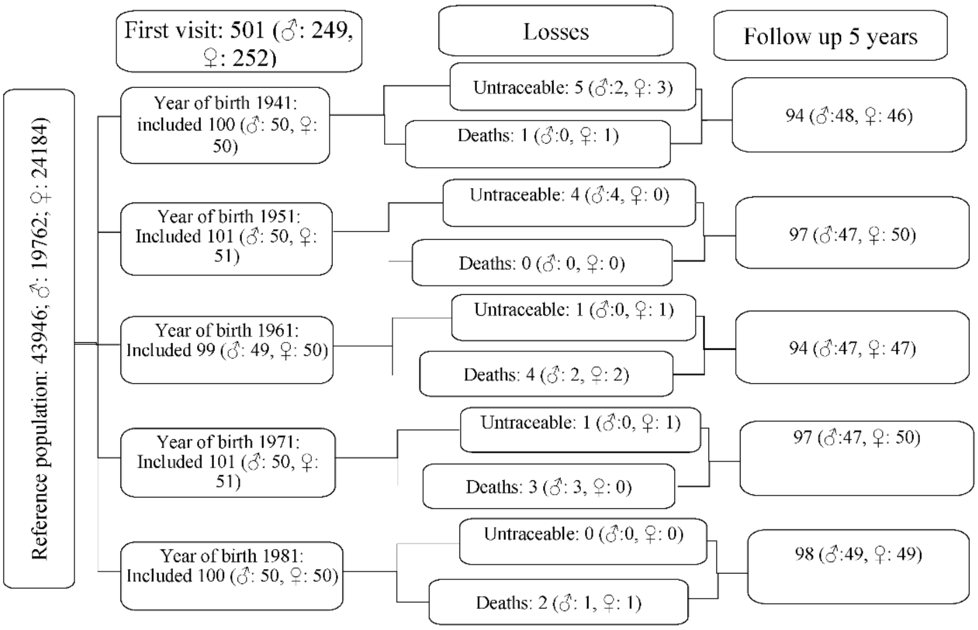

Figure S2. Flowchart of the follow-up phase of the EVA study. During the 5 years of follow-up, 10 subjects died (6♂: men; 4 ♀: women) and we were unable to contact 11 people (6 ♂: men; 5 ♀: women).

Table S1. Differences between the subjects analyzed, those who died and those lost during follow-up.

	Global (n= 501)	Exitus (n=10)	Lost (n=11)	P value
**Lifestyles**				
Alcohol, (g/W)	91.17±85.78	91.50±171.82	28,18±39.20	0.138
Smoking index	21.18±22.08	16.22±16.04	15.15±8.78	0,745
MD, (total score)	7.17±2.07	6.50±1.716	6.82±2.40	0,525
Total PA, (METs/m/W)	2536±3307	1848±1456	2782±3329	0,780
Tiempo sentado (Horas/W)	42.18±17.80	41.19±15.	41.88±19.51	0,984
**Conventional risk factors**				
Age, (years)	56±14.20	65±11.97	44±9.82	0,002
SBP, (mmHg)	120.80±23.38	127.05±14.51	120.80±23.39	0,217
DBP, (mmHg)	75.63±9.93	77.65±15.46	69.05±10.07	0,081
Total cholesterol, (mg/dl)	195.10±32.84	193.30±28.04	181.55±15.19	0,389
LDL cholesterol, (mg/dl)	115.53±29.48	125.30±32.72	105.36±17.97	0,298
HDL cholesterol, (mg/dl)	58.94±16.26	49.90±15.95	58.73±9.13	0,216
Triglycerides, (mg/dl)	103.18±53.84	115.00±41.90	86.91±25.52	0,468
FPG, (mg/dl)	88.03±16.75	106.40±35.34	79.55±9.64	0,001
HbA1c, (%)	5.48±0.55	5.73±0.95	5.30±0.24	0,207
Height, cm	165,15±9.77	162.50±6.29	165.73±8.39	0,678
Weight, kg	62.71±13.78	69.75±8.84	65.82±6.51	0,216
BMI, (kg/m^2)	26.57±4.25	26.53±4.06	24.00±2.22	0,136
**Arterial stiffness measurements**				
cfPWV, (m/seg)	8.15±2.49	7.64±2.62	6.58±1.18	0,090
baPWV, (m/seg)	12.93±2.68	16.37±3.27	11.45±1.59	<0,001
CAVI	8.01±1.44	9.81±1.04	7.19±0.82	<0,001
CAIx	26.84±12.79	35.38±8.59	15.27±10.60	0,002

Values are means ± standard deviations for continuous data and number and proportions for categorical data.

p value: differences between men and women.

Adherence MD. SD. standard deviation; gr/W. grams/week; FA. physical activity; METs/m/W. basal metabolic rate/minute/week; MD. mediterranean diet; SBP. systolic blood pressure; DBP. diastolic blood pressure; LDL. low–density lipoprotein; HDL. high–density lipoprotein; FPG. fasting plasma glucosa; HbA1c. glycosylated hemoglobin; BMI. body mass index; cfPWV: Carotide-femoral pulse wave velocity; baPWV: Brachial-Ankle pulse wave velocity; CAVI: Cardiac-ankle vascular Index; CAIx: Central augmentation index.

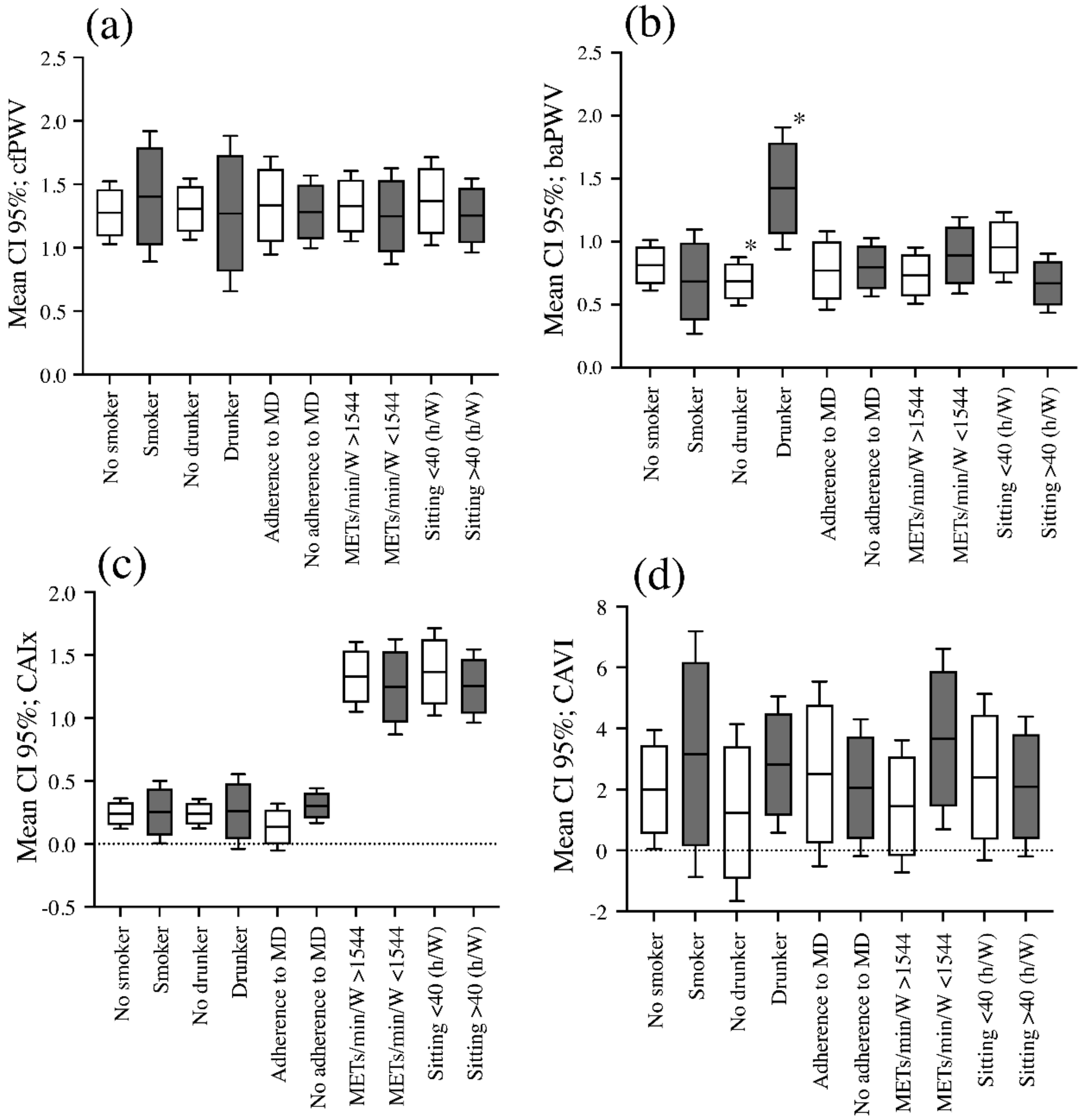

Figure S3: Increase of estimated arterial stiffness measures according to healthy or unhealthy lifestyle in men. (a) cfPWV (b) baPWV (c) CAIx (d) CAVI. CI: confidence interval; MD: Mediterranean Diet; METs/min/W: basal metabolic rate/minutes/week; h/W: hours/week. cfPWV: Carotide-femoral pulse wave velocity; baPWV: Brachial-Ankle pulse wave velocity; CAVI: Cardiac-ankle vascular Index; CAIx: Central augmentation index.
*: $p<0.05$.

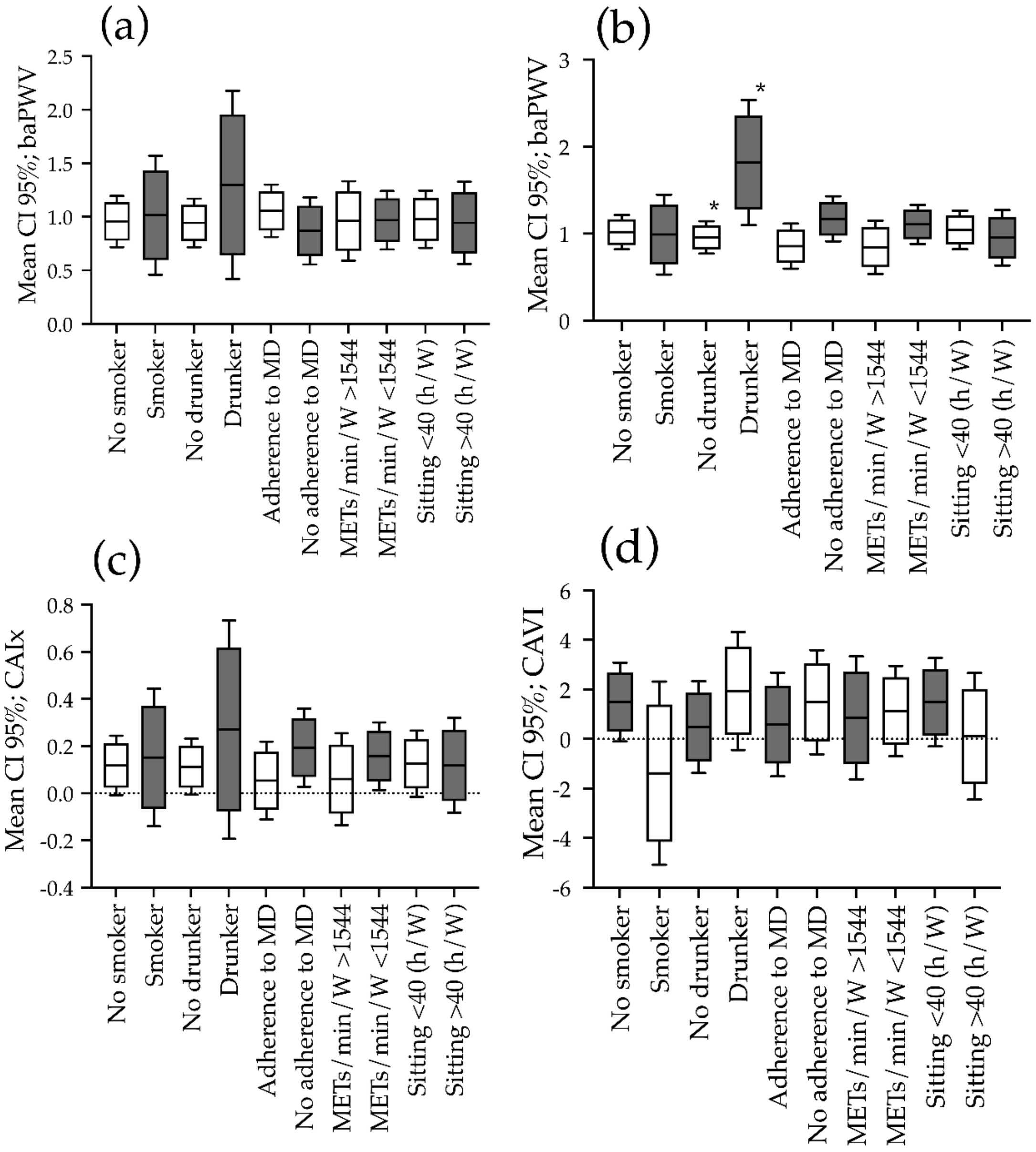

Figure S4: Increase of estimated arterial stiffness measures according to healthy or unhealthy lifestyle in women. (a) cfPWV (b) baPWV (c) CAIx (d) CAVI. CI: confidence interval; MD: Mediterranean Diet; METs/min/W: basal metabolic rate/minutes/week; h/W: hours/week. cfPWV: Carotide-femoral pulse wave velocity; baPWV: Brachial-Ankle pulse wave velocity; CAVI: Cardiac-ankle vascular Index; CAIx: Central augmentation index.

*: $p<0.05$.

Table S2. Association of increasing arterial stiffness with lifestyles in men. Multiple regression analysis.

**cfPWV. (m/s)**	β	**IC 95%**	p
Tobacco index	0.005	(-0.014 to 0.025)	0.607
Alcohol consumption (gr/W)	0.001	(-0.002 to 0.004)	0.522
Mediterranean Diet (total score)	-0.024	(-0.149 to 0.100)	0.699
Total PA (METs/min/W)	0.015	(-0.045 to 0.075)	0.614
Sitting (h/W)	-0.002	(-0.016 to 0.012)	0.768
**baPWV. (m/s)**			
Tobacco index	0.017	(0.001 to 0.0032)	0.032
Alcohol consumption (gr/W)	0.004	(0.001 to 0.007)	0.003
Mediterranean Diet (total score)	0.001	(-0.009 to 0.100)	0.990
Total PA (METs/min/W)	-0.008	(-0.056 to 0.040)	0.754
Sitting (h/W)	-0.006	(-0.017 to 0.005)	0.298
**CAVI**			
Tobacco index	-0.001	(-0.013 to 0.011)	0.897
Alcohol consumption (gr/W)	0.000	(-0.002 to 0.001)	0.762
Mediterranean Diet (total score)	-0.051	(-0.110 t 0.009)	0.094
Total PA (METs/min/W)	-0.006	(-0.035 to 0.022)	0.660
Sitting (h/W)	0.006	(-0.001 to 0.012)	0.079
**CAIx75**			
Tobacco index	0.121	(-0.065 to 0.307)	0.199
Alcohol consumption (gr/W)	0.014	(-0.007 to 0.035)	0.183
Mediterranean Diet (total score)	0.362	(-0.615 to 1.340)	0.466
Total PA (METs/min/W)	-0.049	(-0.521 to 0.424)	0.839
Sitting (h/W)	-0.012	(-0.119 to 0.094)	0.819

Multiple regression analysis using cfPWV, baPWV, CAVI and CAIxI as dependent variables. As independent variables lifestyles (Tobacco consumption. alcohol. Mediterranean diet score. Total physical activity. hours sitting per week) and as adjustment variables age, mean arterial pressure and consumption of antihypertensive drugs. hypoglycemic and lipid-lowering agents.

cfPWV: Carotide-femoral pulse wave velocity; baPWV: Brachial-Ankle pulse wave velocity; CAVI: Cardiac-ankle vascular Index; CAIx: Central augmentation index. m/s: metres/second; gr/W: grames per week; PA: Physical Activity; METs/m/W: basal metabolic rate/minute/week; h/W: hours/week.

Table S3. Association of increasing arterial stiffness with lifestyles in women. Multiple regression analysis.

**cfPWV. (m/s)**	β	IC 95%	p
Tobacco index	0.006	(-0.005 to 0.017)	0.292
Alcohol consumption (gr/W)	0.000	(-0.005 to 0.006)	0.959
Mediterranean Diet (total score)	-0.030	(-0.145 to 0.077)	0.543
Total PA (METs/min/W)	0.015	(-0.118 to 0.057)	0.495
Tobacco index	-0.003	(-0.016 to 0.009)	0.621
**baPWV. (m/s)**			
Tobacco index	0.003	(-0.008 to 0.014)	0.565
Alcohol consumption (gr/W)	0.009	(0.004 to 0.014)	0.001
Mediterranean Diet (total score)	-0.112	(-0.204 to -0.021)	0.017
Total PA (METs/min/W)	-0.029	(-01.03 to 0.044)	0.428
Tobacco index	-0.004	(-0.014 to 0.007)	0.486
**CAVI**			
Tobacco index	0.003	(-0.004 to 0.011)	0.396
Alcohol consumption (gr/W)	0.001	(-0.002 to 0.005)	0.445
Mediterranean Diet (total score)	-0.043	(-0.102 to 0.015)	0.148
Total PA (METs/min/W)	-0.040	(-0.086 to 0.006)	0.091
Tobacco index	0.000	(-0.006 to 0.007)	0.947
**CAIx75**			
Tobacco index	0.049	(-0.041 to 0.139)	0.279
Alcohol consumption (gr/W)	-0.012	(-0.063 to 0.040)	0.656
Mediterranean Diet (total score)	0.172	(-0.577 to 0.921)	0.651
Total PA (METs/min/W)	0.014	(-0.576 to 0.604)	0.962
Tobacco index	-0.014	(-0.099 to 0.072)	0.753

Multiple regression analysis using cfPWV, baPWV, CAVI and CAIxI as dependent variables. As independent variables lifestyles (Tobacco consumption. alcohol. Mediterranean diet score. Total physical activity. hours sitting per week). Adjusted variables are age, mean arterial pressure and consumption of antihypertensive drugs. hypoglycemic and lipid-lowering agents.

cfPWV: Carotide-femoral pulse wave velocity; baPWV: Brachial-Ankle pulse wave velocity; CAVI: Cardiac-ankle vascular Index; CAIx: Central augmentation index. m/s: metres/second; gr/W: grames per week; PA: Physical Activity; METs/m/W: basal metabolic rate/minute/week; h/W: hours/week.

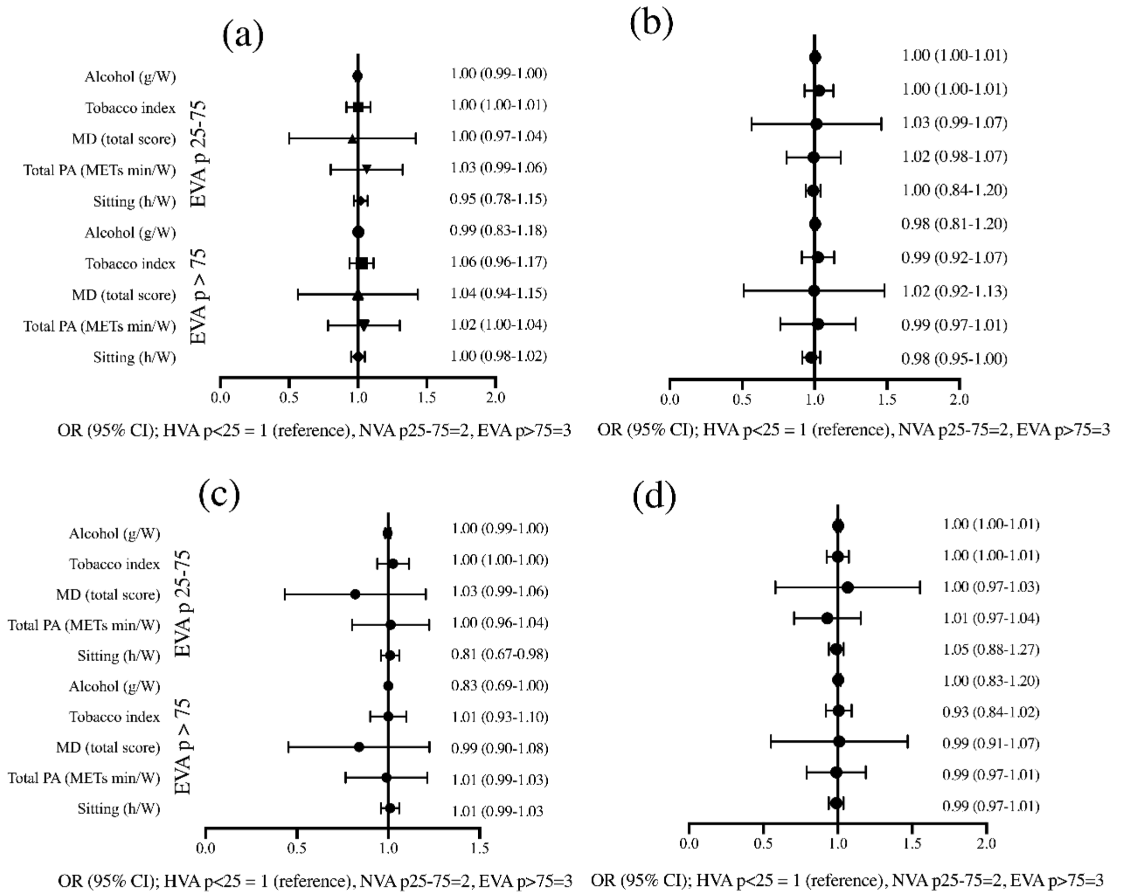

Figure S5: Association of lifestyles with increasing of stiffness measures values in men. Multinomial logistic regression analysis. (a) using cfPWV (b) using baPWV (c) using CAIx (d) using CAVI como variables dependientes. Consumo de tabaco, de alcohol, score de dieta meditermea. Actividad fsica total and horas sentado a la semana como variables independientes y como variables de ajuste la edad, la presin arterial media y el consumo de frmacos hipotensores. hipoglucemiantes e hipolipemiantes.
cfPWV: Carotide-femoral pulse wave velocity; baPWV: Brachial-Ankle pulse wave velocity; CAVI: Cardiac-ankle vascular Index; CAIx: Central augmentation index; g/W: grames per week; MD: Mediterranean diet; PA: Physical Activity; METs/min/W: basal metabolic rate/minutes/week; h/W: hours/week; OR: odd ratio; HVA: healthy vascular ageing; NVA: normal vascular ageing; EVA: early vascular ageing.

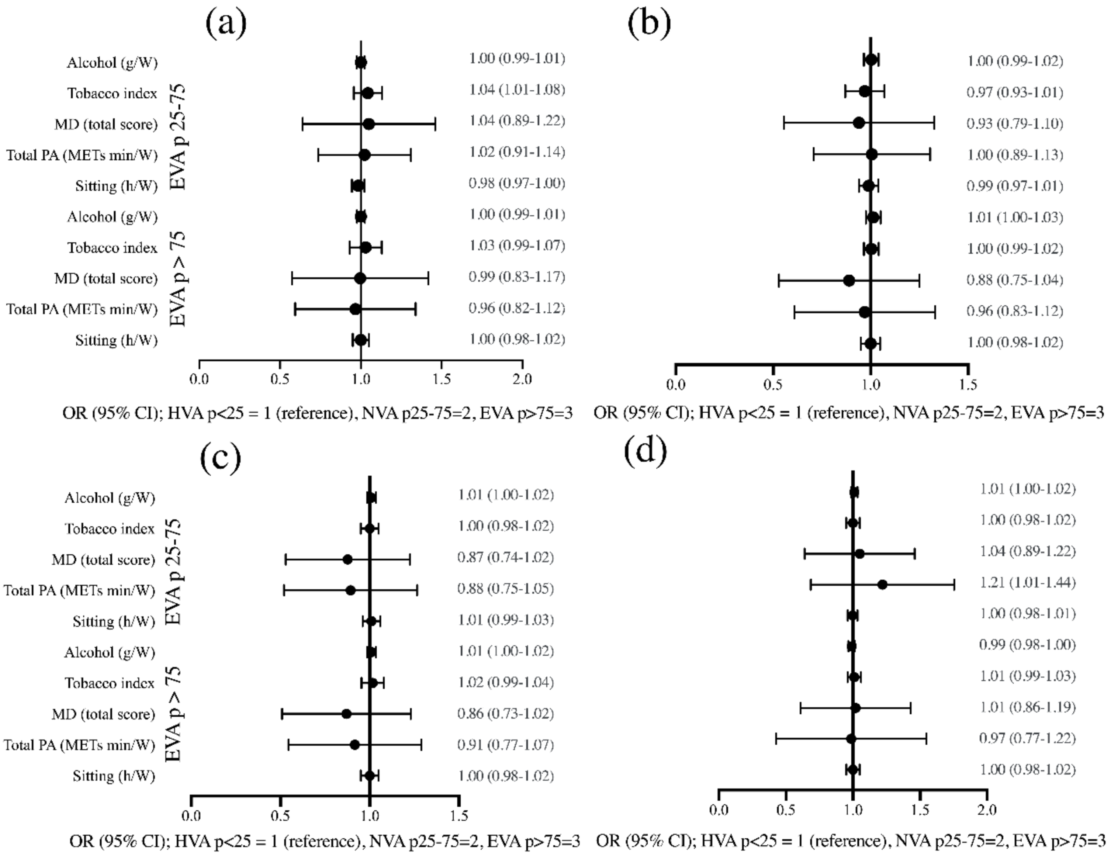

Figure S6: Association of lifestyles with increasing of stiffness measures values in women. Multinomial logistic regression analysis. (a) using cfPWV (b) using baPWV (c) using CAIx (d) using CAVI como variables dependientes. Consumo de tabaco, de alcohol, score de dieta meditermea. Actividad fsica total and horas sentado a la semana como variables independientes y como variables de ajuste la edad, la presin arterial media y el consumo de frmacos hipotensores. hipoglucemiantes e hipolipemiantes.

cfPWV: Carotide-femoral pulse wave velocity; baPWV: Brachial-Ankle pulse wave velocity; CAVI: Cardiac-ankle vascular Index; CAIx: Central augmentation index; g/W: grames per week; MD: Mediterranean diet; PA: Physical Activity; METs/min/W: basal metabolic rate/minutes/week; h/W: hours/week; OR: odd ratio; HVA: healthy vascular ageing; NVA: normal vascular ageing; EVA: early vascular ageing.
